# Feasibility of Recruiting a Diverse Sample of Men Who Have Sex with Men: Observation from Nanjing, China

**DOI:** 10.1371/journal.pone.0077645

**Published:** 2013-11-14

**Authors:** Weiming Tang, Haitao Yang, Tanmay Mahapatra, Xiping Huan, Hongjing Yan, Jianjun Li, Gengfeng Fu, Jinkou Zhao, Roger Detels

**Affiliations:** 1 Department of Epidemiology, School of Public Health, University of California Los Angeles, Los Angeles, California, United States of America; 2 Jiangsu Provincial Center for Disease Control and Prevention, Nanjing, Jiangsu, China; 3 Impact Results and Evaluation Department, The Global Fund to Fight AIDS, Tuberculosis and Malaria, Geneva, Switzerland; University of Washington, United States of America

## Abstract

**Background:**

Respondent-driven-sampling (RDS) has well been recognized as a method for sampling from most hard-to-reach populations like commercial sex workers, drug users and men who have sex with men. However the feasibility of this sampling strategy in terms of recruiting a diverse spectrum of these hidden populations has not been understood well yet in developing countries.

**Methods:**

In a cross sectional study in Nanjing city of Jiangsu province of China, 430 MSM were recruited including 9 seeds in 14 weeks of study period using RDS. Information regarding socio-demographic characteristics and sexual risk behavior were collected and testing was done for HIV and syphilis. Duration, completion, participant characteristics and the equilibrium of key factors were used for assessing feasibility of RDS. Homophily of key variables, socio-demographic distribution and social network size were used as the indicators of diversity.

**Results:**

In the study sample, adjusted HIV and syphilis prevalence were 6.6% and 14.6% respectively. Majority (96.3%) of the participants were recruited by members of their own social network. Although there was a tendency for recruitment within the same self-identified group (homosexuals recruited 60.0% homosexuals), considerable cross-group recruitment (bisexuals recruited 52.3% homosexuals) was also seen. Homophily of the self-identified sexual orientations was 0.111 for homosexuals. Upon completion of the recruitment process, participant characteristics and the equilibrium of key factors indicated that RDS was feasible for sampling MSM in Nanjing. Participants recruited by RDS were found to be diverse after assessing the homophily of key variables in successive waves of recruitment, the proportion of characteristics after reaching equilibrium and the social network size. The observed design effects were nearly the same or even better than the theoretical design effect of 2.

**Conclusion:**

RDS was found to be an efficient and feasible sampling method for recruiting a diverse sample of MSM in a reasonable time.

## Introduction

While conducting a research involving hard-to-reach populations, such as injecting drug users (IDUs), commercial sex workers (CSWs) or men who have sex with men (MSM), ensuring the representativeness of the study sample is a big challenge. In the past two decades, many strategies have been used to recruit diverse samples from these hidden population groups, but generalizability of the study results always remained an issue and selection bias was always a probability. These recruitment methods included time-location sampling, snowballing and targeted sampling. However, most of these methods only provided limited coverage and could only claim pooled representation of the target populations [1]. To deal with these problems, Heckathorn DD introduced respondent-driven sampling (RDS) in 1994, while studying HIV-related risk behaviors among IDUs residing in the eastern part of United States [2].

In the past few years, RDS has been widely used in many countries to recruit hard-to-reach populations and to conduct large-scale HIV serologic and behavioral surveys [3]. It has also been recognized and adopted by public health researchers as a promising alternative method for sampling from most-at-risk populations [4]–[9]. RDS is a variant of the chain-referral methodology, which requires that the population of interest be internally well-connected though social networks [2], [9]. The method uses a mathematical model to compensate for the non-representativeness by keeping track of the recruitment process and using probabilistic weights. While sampling from a hidden population from which recruitment of a random sample is not feasible otherwise, the initially selected participants (“Seeds”) in RDS need not be random to have a somewhat representative sample as long as the seeds belong to target population. With the continuation of the recruitment process in RDS, the distribution of the characteristics of the sample stabilizes gradually and this condition is termed as “reaching the equilibrium” [2], [10], [11]. These frameworks of RDS help in minimizing the sampling biases found commonly in chain referral sampling [2], [9]. However, the feasibility of RDS still remained unclear.

MSM population is probably playing a significant role in the HIV epidemic of China where like other countries in Asia, HIV sero-positivity level is rising among MSM. Nanjing, a metropolitan city, located in one of the largest economic zone of China and having a population of approximately 8 million (according to 2010 census) is no exception. HIV percentage positivity among the participating MSM in Nanjing has increased from none detected in 2003 to 5.8% in 2007 [12]. “Money boy” is a group of male who provide commercial sexual services to other male in different parts of China and some other countries. In Nanjing, commercial sex with money boys is very common among MSM population, especially among young males [13], [14].

Several studies have been conducted to assess whether RDS is a feasible strategy to recruit samples from hidden populations [5], [7], [8]. However, most of these studies either did not evaluate the efficiency of RDS in terms of reaching a diverse spectrum of hard-to-reach populations or didn't address design effect (Deff) and sample size issues. These limitations called for this study among the MSM population of Nanjing, a large metropolitan city of China, with the aims of assessing the feasibility of RDS for reaching this hard-to-reach population, whether the strategy yields a diverse sample and whether it reaches the designed sample size and design effect.

## Materials and Methods

### Ethics Statement

All the participants provided written informed consent for voluntarily taking part in this survey on their own. Signed informed consent was obtained from each of the participants prior to interview, blood collection and intervention at each round of the surveys. Each of the participants had the ability to decline or withdraw himself from this survey at any time. The questionnaires and written consent document were separately kept in locked cupboards at the study sites and unauthorized persons had no access on them.

The study process and content were approved by the Ethics Committee at Jiangsu Provincial Center for Disease Prevention and Control (JSCDC, Nanjing, Jiangsu 210009, China.

### Study Design and Sampling Methods

In the year 2008 between the months of May and August a cross-sectional study was conducted using RDS as the sampling strategy for recruiting MSM in Nanjing city of China. The sampling began with a set of initial participants (“seeds”) recruited with the help of MSM community-based organizations, operators of bars and bathhouses/spas and from restrooms/parks or internet. 9 individual seeds who were different from each other in terms of income, age, occupation and “cruising area” (venues for meeting sexual partners), were thus recruited. These seeds initiated the expanding chain of referrals, whereby respondents from each link in the chain or “wave” referred other respondents to form the subsequent waves of referral [10]. It has been shown that using RDS any member of a hard-to-reach community can be reached theoretically by using six separate waves (principle of “six degrees of separation”) [10]. In this study, each seed initially recruited three other MSM from their social networks for behavioral evaluation and serological testing, using uniquely numbered coupons to allow tracking of the recruitment process. Each respondent received a gift (containing lubricant and condoms) worth 4.50 USD approximately to compensate for his time contribution. Three recruitment coupons were also given to them to be passed on to their acquaintances. If at least one new participant was recruited with a coupon, the respondent making the referral with that coupon received an additional gift (prepaid phone card) worth 4.50 USD approximately as a token of appreciation of his effort. Official residency, education level, marital status, syphilis sero-status, sexual orientation and cruising area these six key factors were used to monitor whether RDS had reached equilibrium or not.

Homophily is a statistic that describes the mixing patterns in networks and the probability of an HIV-positive individual successfully referring another HIV-positive individual from a population. Homophily can be negative or positive (ranging from −1 to +1), depending on whether an individual preferentially contacts or avoids someone with the same given characteristic [6], [10], [15]–[18]. It has been shown that when homophily is zero for all groups, the estimated population proportion (EPP) for the target population is identical to the actual sample population proportions (SPE) [2], [6], [10], [15]–[19].

To attain the distribution of the sample characteristics free of the biasing effects of the non-random seed selection process, equilibrium distributions were set at the statistical software RDSAT (software for statistical analysis of data from sample recruited by RDS) to fall within 2% of the sample distribution [2], [10], [11]. Social network size was defined as the number of MSM in the city known (familiar with face, name/nickname, had contact information, and could get in touch with him in the next month) to the participant. The design effect was determined by the ratio of the actual variance under the used sampling method and the variance computed under the assumption of simple random sampling [19], [20].

The study was conducted at the clinic for sexually transmitted infections (STI) of the Center for Disease Control and Prevention of Jiangsu Province (JSCDC) between May and August, 2008. Eligibility criteria for the participants were: 1) male; 2) having sex with men (oral and/or anal) within the past year; 3)18 years or older; 4) had not participated in a similar survey within the past three months and 5) had a valid referral coupon.

### Measures

#### Data measures

Duration of survey, completion of the recruitment process using RDS, characteristics of the seeds and their recruits and the equilibrium of the key factors were used as the parameters for the evaluation of the feasibility of RDS. While doing this design effects (Deff) of selected variables were also determined. Homophily of key variables, the proportional distribution of selected demographic variables and the social network size were the indicators to assess the diversity of RDS.

During data collection, the process of distribution of the referral coupons was kept restricted to control exponential sample growth. The number of distributed coupons was reduced from three to two and then to one after the sample size reached 350, and no more coupons were distributed after the sample size reached 420.

Face-to-face interviews with a structured questionnaire were conducted to collect information on recruitment patterns, demographics, HIV knowledge, coverage of HIV prevention services, recent sexual behavior and drug use, and STI-related symptoms. Demographic information included age (less than 20, 20–29, 30–39, 40–49, and over 50 years old), marital status (single, married and divorced/widowed), occupation (15 occupational categories that included most recognized occupations), education level (illiterate, elementary school, junior high school; senior high school, technical secondary school and junior college/college degree/higher), residency (official resident of Jiangsu province or other provinces) and family income in Yuan (less than 2,000, 2001–3000, 3001–4000, 4001–5000, and more than 5000; 1 USD = approximately 6.4 Yuan).

Self-identified sexual orientations were classified as undecided, only homosexual, mainly heterosexual and bisexual. Equilibrium distributions were assessed based on sexual orientation, being single, official residency for Nanjing, college degree or higher education, proportion of syphilis sero-positive cases and recruiting sexual partners online. Homophilies were also calculated for self-identified sexual orientation and proportion of syphilis cases.

#### Serologic measures

Five ml of venous blood was collected from each subject for HIV and syphilis testing. HIV antibodies were screened using a rapid test (Acon Biotech Co., Ltd, lot 200803973/WB). If the screening result was positive, it was confirmed by Western blot (HIVBLOT 2.2, Genelabs Diagnostics, Singapore, lot AE8039). Syphilis antibodies were screened using Rapid Plasma Reagin (RPR; Beijing WanTai Biological Pharmacy Enterprise Co., Ltd., lot N20080404) test and confirmed with Treponema Pallidum Particle Agglutination assay (TPPA; Livzon Group Reagent Factory, Guangdong, China, lot VN80803). Syphilis positivity was deemed “current” when both TPPA and RPR assays were positive.

### Sample size and design effect (Deff)

Using Deff = 2.0, for the detection of a 10% increase in high risk sexual behavior with 80% power and 95% confidence level, the required sample size for this study was calculated to be 460. The detailed sample size estimation process has been described in Appendix S1. In our study, 430 participants were recruited during 14 weeks of study period, including the nine seeds. The Deff for being HIV-negative was 2.48, syphilis-negative 1.87, engaged in unprotected anal sex 2.20, only homosexual 1.85, and having college degree or higher education 2.95. The detailed estimation process for standard error (se), variance using RDS and observed Deff has been described in Appendix S2.

### Data Analyses

Respondent Driven Sampling Analysis Tool (RDSAT), version 5.6 (available free online http://www.respondentdrivensampling.org) was initially used to calculate the population adjusted point estimate, 95% confidence interval and level of homophily. RDS uses network information to account for potential sources of bias in the sample and provides mathematical methods for adjusting estimates based on these biases [21]. Hence the data gathered using RDS methods in this study were analyzed with RDSAT using weighting based on the inverse probability of selection proportional to the size of the network of each participant to adjust for the potential sampling biases. We also used STATA 10.0 to calculate the equilibrium and Deff.

## Results

Among the 430 participants recruited in this study with the help of the 9 seeds, 20 were HIV positive, with crude prevalence of 4.6% and adjusted prevalence of 6.6% (adjusted by RDSAT, based on the weight of network sizes). The characteristics of the 9 seeds for this study are presented in Table 1.

**Table 1:** Seed characteristics and success of recruitment using RDS.

Seed ID	Main venue for meeting partners	Age	Network size	Self-identified sexual orientation	Maximum number of waves	Maximum number of recruits (excepting seeds)	Percent of total participants recruited
A	Bathhouses/spas	44	100	Only Homosexual	6	13	3.1
B	Internet	26	50	Only Homosexual	8	87	20.7
C	Internet	28	35	Only Homosexual	9	65	15.4
D	Restrooms/parks	24	10	Bisexual	3	11	2.6
E	Bathhouses/spas	38	20	Bisexual	13	73	17.3
F	Restrooms/parks	39	2	Only Homosexual	0	0	0.0
G	Bars	41	100	Bisexual	11	86	20.4
H	Bars	32	60	Only Homosexual	11	83	19.7
I	Internet	35	50	Only Homosexual	1	3	0.7

### Feasibility

Among the 455 respondents who visited the study site and provided consent, 25 did not have a valid recruitment coupon and were excluded from the study. Data from the other 430, including the 9 seeds, were collected over a period of 14 weeks (between 5^th^ May and 1^st^ August, 2008). The survey office was open six days a week from 8:30 am to 5:00 pm and two to four interviewers were present at the site during this period. The average number of completed interviews was 30.71(minimum = 2, maximum = 64) per week. The wait time for being interviewed and seen by the clinicians was less than 20 minutes and the average time that a participant spent at the survey site was about 45 minutes

**Figure 1:**
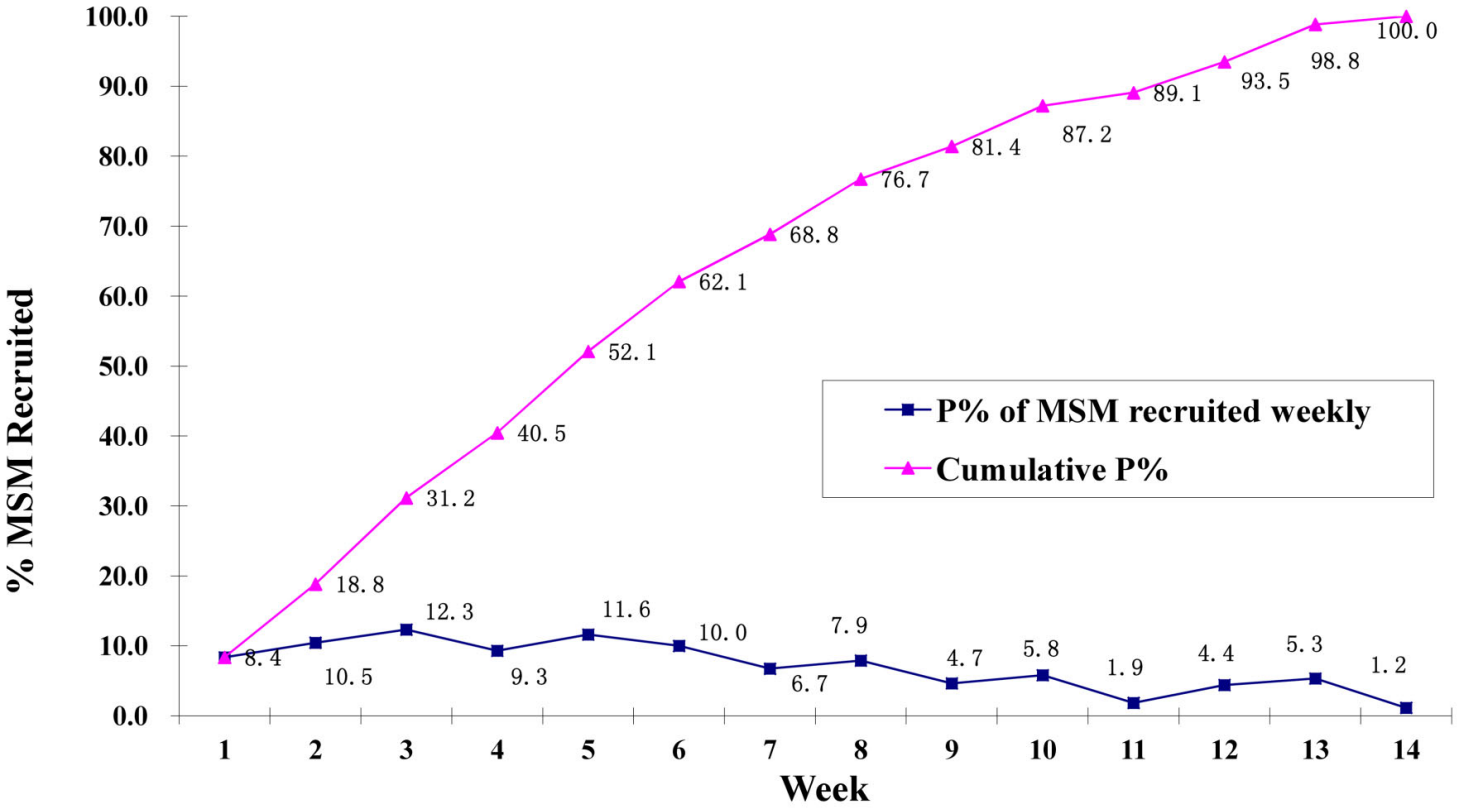
Flow of study participants over the course of 14 weeks, Nanjing, China, 2008.

Figure 1 shows the recruitment flow of the participants (level of enrollment) as the survey progressed toward the targeted sample size. As the process of recruitment progressed beyond the 5^th^ week, the percentage of participants coming to the survey site started to decrease. When the number of recruited subjects reached 350, we gradually reduced the number of recruitment coupons given to participants from 3 to 2 and then to 1 while the coupon validity was shortened from 15 to 10 days. After the recruitment of 420 subjects, no more coupons were distributed. By the end of 14^th^ week, the survey reached the sample size of 430.

To see how coupons were distributed by the participants within their social networks, participants were asked about their relationship with the person who gave them the coupon. 41.6% received coupons from a close friend, 3.0% from a sexual partner more than six months ago and 13.3% from a sexual partner within the past six months. Only 3.7% reported receiving one from a stranger. The majority (90.2%) of participants reported that their primary reason for accepting a coupon and coming to the clinic site was to be tested and treated for HIV/STIs. Only 0.3% reported coming only for the incentive.

An equilibrium distribution system was used to evaluate whether the recruitment met the design needs. The rate of syphilis reached equilibrium by the fifth wave. Equilibrium distribution for being self-identified homosexual, single and resident of Nanjing were all reached by the seventh wave while usually meeting sexual partners on the internet and having a college degree or higher education were reached by the tenth wave.

### Diversity

56.8% of the participants found their sexual partners on the internet, 16.2% at pubs, discos, tearooms or clubs; 15.2% at spas, bathhouses, saunas or massage centers; 4.3% in parks and public restrooms while 7.6% found them somewhere else (Table 2).

**Table 2:** Venues for meeting sexual partners among participants and recruiter.

Venue of person giving referral coupon	Venue of recruiter											
	Bars		Bathhouse		Park		Internet		Others		Total	
	N	%	N	%	N	%	N	%	N	%	N	%
Bars	32	42.7	12	16.0	1	1.3	26	34.7	4	5.3	75	100
Bathhouse	10	15.9	30	47.6	3	4.8	10	15.9	10	15.9	63	
Park	1	4.8	6	28.6	3	14.3	8	38.1	3	14.3	21	100
Internet	25	10.1	10	4.0	9	3.6	188	76.2	15	6.1	247	100
Others	0	0.0	6	40.0	2	13.3	7	46.7	0	0.0	15	100
Total	68	16.2	64	15.2	18	4.3	239	56.8	32	7.6	421	100

Indicators of recruitment	Bars	Bathhouse	Park	Internet	Others	Total	
Sample proportion (%)	16.20	15.20	4.30	56.80	7.60	100	
Equilibrium proportion (%)	14.80	17.30	4.70	55.50	7.60	100	
RDS-adjusted proportion, P (%)	13.90	31.10	4.50	44.20	6.30	100	
Standard error of P	0.026	0.045	0.011	0.043	0.011		
Homophily	0.325	0.24	0.103	0.57	−1		
Estimated network size	6.8	3.5	6.7	8	7.7		

N =  Number of subjects.

Table 2 also presents the joint recruitment patterns of the venues for meeting sexual partners. The homophily of this variable is not close to zero (0.325 for bars, 0.240 for bathhouse, 0.103 for parks, 0.57 for internet and −1.0 for others).

In our study, 5.0% participants reported that they at least once paid for sex in the past six months, and another 5.8% participants reported that they were paid for sex in the past six months. Meanwhile, 2.6% participants self reported that they were drug users.

**Figure 2:**
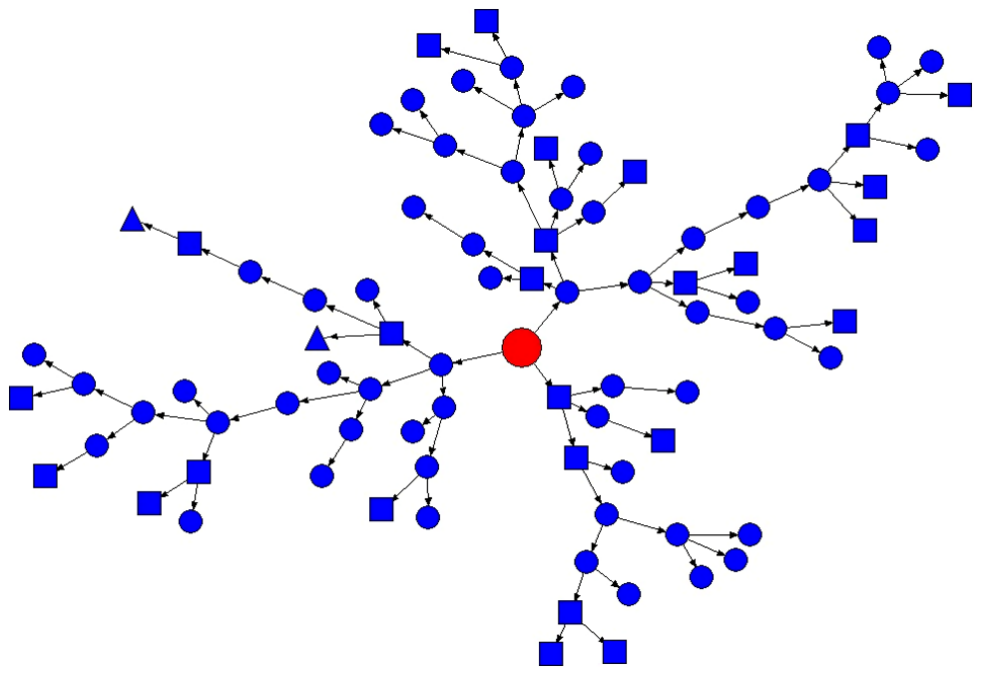
Branching patterns (NetDraw) recruited by most effective seed. Legends: Circle for “Undecided”, square for “Homosexual” and triangle for “Bisexual”.

Figure 2 shows the branching patterns of the path of maximum recruitment (88 recruits, 8 waves), which began with one self-identified homosexual seed. The figure also shows the cross distributions of sexual orientations among different chains.

The homophily of the self-identified sexual orientations was 0.111 for only homosexuals (n = 242), indicating that only homosexuals were probably socially insular and preferentially recruited other self-identified homosexuals more (59.7%) than MSM of other orientations (40.3%). Bisexuals (n = 167) demonstrated a homophily of −0.012 and mainly heterosexuals (n = 10) demonstrated a homophily of 0.236.

Table 3 shows the RDS estimates for the proportion of syphilis cases among participants. MSM infected with syphilis (n = 54) demonstrated a homophily of 0.077 while those who were syphilis-negative (n = 376) demonstrated a homophily of 0.251. Although only 14.6% of the population was syphilis-positive, they recruited 21.2% other syphilis cases and 78.8% syphilis-negatives. While 85.4% of the population was syphilis-negative, they recruited 89.1% syphilis-negative and 10.9% positive participants. However, cross-recruitment was substantial (19.2%), implying that recruitment chains did not become trapped within a single group, but instead crossed lines. This contributed toward a strong convergence between the sample composition (12.6% syphilis cases) and the equilibrium sample composition (12.1% syphilis cases). It also indicated that homophily and estimated network size both were potentially important factors for the referral process. Average network size was found to be 6.9 and 5.6 for syphilis negative and positive participants respectively.

**Table 3:** Characteristics of the estimation process using RDS, regarding syphilis serostatus.

Participants giving referral coupons	Syphilis status of subsequent recruit	
	Infected	Uninfected
Syphilis-positive participants		
Recruitment count	11	41
Recruitment proportion (%)	21.2	78.8
Syphilis-negative participants		
Recruitment count	40	328
Recruitment proportion (%)	10.9	89.1
Estimated network size	5.6	6.9
Sample proportion, P_s_	12.6	87.4
RDS-adjusted proportion, P	14.6	85.4
Equilibrium proportion, P_e_	12.1	87.9
Absolute discrepancy between P_s_ and P_e_	0.5	
Standard error of P	2.8	2.8
RDS-weight	1.159	0.977
Homophily	0.077	0.251

The recruitment patterns according to self-identified sexual orientation indicated mixing of different orientations amongst participants (Table 4). However, there was a tendency for recruitment within a group, along with considerable cross-group recruitment. Overall, homosexuals recruited 60.0% only homosexuals, 1.2% mainly heterosexuals, 36.8% bisexuals. Bisexuals recruited 40.5% bisexuals, 52.3% only homosexuals. There was a strong convergence between the sample composition (56.8% only homosexuals, 2.4% mainly heterosexuals, 38.2% bisexuals) and the equilibrium sample composition (56.7% only homosexuals, 2.6% mainly heterosexuals, 38.1% bisexuals). Homophily for self-identified sexual orientation was 0.111 in cases of only homosexuals, 0.236 for mainly heterosexuals and −0.012 for bisexuals. Mainly heterosexuals had a larger network size (9.3) compared to only homosexuals (7.0) and bisexuals (6.2). When the equilibrium distribution was reached, the proportions of self-identified sexual orientations were 56.4% only homosexual, 38.8% bisexual, 2.2% mainly heterosexual.

**Table 4:** Sexual orientations of the participants.

Sexual orientation of person giving referral coupon	The identified sexual status of recruiter								
	Only Homosexuals		Mainly Heterosexuals		Bisexual		Undecided		Total
	Number	%	Number	%	Number	%	Number	%	
Only Homosexuals	153	60.0	3	1.2	94	36.8	5	2.0	255
Mainly Heterosexuals	1	12.5	2	25.0	5	67.5	0	0.0	8
Bisexuals	80	52.3	5	3.3	62	40.5	6	3.9	153
Other	5	100.0	0	0.0	0	0.0	0	0.0	5
Total	239	56.8	10	2.4	161	38.2	11	2.6	421

Indicators of recruitment	Only Homosexuals	Mainly Heterosexuals	Bisexual	Undecided
Sample proportion (%)	56.8	2.4	38.2	2.6
Equilibrium proportion (%)	56.7	2.6	38.1	2.6
RDS-adjusted proportion, P (%)	54.7	1.8	41.0	2.5
Standard error of P	0.033	0.008	0.031	0.007
Homophily	0.111	0.236	−0.012	−1.000
Estimated network size	7.0	9.3	6.2	7.1

At equilibrium, 11.4% were aged less than 20 years, 58.6% belonged to 20–30 years, 17.9% 30–40 years, 10.2% 40–50 years, and 1.9% over 50. Equilibrium proportions for the average monthly income in Yuan were 31.8% under 1,000, 24.0% 1,000–2,000, and 12.8% over 4,000.

The final sampling shows the results for social network sizes. The proportions of social network sizes were 27.4% under six (adjusted proportion 67.4%, 95% CI 62.1–72.7), 6.0% 6–10 (adjusted proportion 20.1%, 95% CI 16.2–24.0), 25.8% 11–20 (adjusted proportion 9.6%, 95% CI 7.7–11.7), and 20.1% over 20 (adjusted proportion 2.8%, 95% CI 2.1–3.6). The average social network size was 20, the median was 10, the maximum was 400, and the minimum was one. The average social network sizes were 24 for homosexuals, 16 for heterosexuals, 16 for heterosexuals.

Table 5 shows the sample size and design effects of certain variables.

**Table 5:** Estimated design effects of certain variables.

Study description		Study results			
N	Characteristic	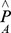	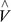 (RDS, 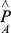 )	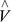 (SRS*, 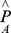 )	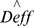 ( 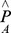 )
430	HIV+	0.066	0.00036	0.00014	2.57
430	Syphilis+	0.146	0.00062	0.00029	2.14
430	Unprotected anal sex	0.705	0.00107	0.00048	2.23
430	Homosexual	0.548	0.00107	0.00058	1.84
430	College degree or higher	0.583	0.00167	0.00056	2.98

*SRS refers to simple random sampling.*

*Note: the formula for estimation are presented in Appendix* ***S2*** *.*

## Discussion

Our study began with nine seeds, recruited a sample of total 430 eligible participants, and observed the crude and adjusted HIV prevalence of 4.6% and 6.6% respectively. The unadjusted prevalence was nearly the same as observed in a study in 2007, using non-probability sampling (convenience sampling) techniques, with HIV prevalence of 4.7% [22]. After adjustment, the HIV prevalence in our study changed by about 43%. Several potential reasons could have lead to this difference: first, the prevalence of HIV was not high, a slight change of the weight might have significantly changed the difference of the two; second, our study might still have the problem of in-group affiliation (e.g. the large homophily for venues where the participants were meeting their partners), which could have biased the crude HIV prevalence, and the RDSAT may potentially eliminated or reduced this bias.

The recruitment period lasted over 14 weeks, with a maximum wave of 14 and recruited participants with varying self-identified sexual orientations who looked for partners in varying venues and the cost of recruitment was acceptable. The nine seeds for our study were selected non-randomly, based on their differing sexual orientations, cruising areas and ages. However, the findings of our study suggested that using RDS with a small number of seeds recruited from non-random, well-defined venues, successful recruitment of participants from a broader spectrum was feasible. This reflected the Markov theory that biases introduced into a chain referral sample by the non-random selection of an individual (seed) are weakened with each recruitment wave and are ultimately eliminated [2], [19], [23]. As recruitment continued, the participants were gradually more distant from the seeds who led to the recruitment and the recruitment ran by itself for at least some if not all of the seeds.

An important character of RDS is that if it is successful, RDS could reach the most hard-to-reach population, like drug users and money boy, by capturing individuals in hidden groups from the social network of the same group. The results of our study demonstrated such ability of RDS. In our study, we found that about 5.0%, 5.8% and 2.6% participants had paid for sex in last six months, were paid for sex during last six months and used drug in the past year respectively. Such findings further supported the diversity of the participants in our study.

We found that the majority of the participants were recruited by someone in their own personal social network (96.3%). The majority (90%) responded because they wanted to be tested and treated (if required) for HIV, syphilis and HCV. While we accept that the diversity demonstrated in our study did not ensure representativeness, due to the fact that there was always a higher likelihood for selection of more concerned and compliant participants, we still claim that offering HIV/STI testing and treatment brought forth more participants from this hidden population.

The variables of interest reached equilibrium and a relatively small number of waves were needed to yield sufficient sociometric depth to attain an equilibrium distribution. To reach the equilibrium of self-identified sexual orientation, marital status, residency, proportion of syphilis-positives and education levels, we needed to enroll seven, seven, six, seven, five, and ten waves, respectively. Reaching equilibrium also confirmed the uniformity of the seeds who remained in the study, which ensured more internal validity. From these results, we concluded that after adequate waves, reaching equilibrium in recruitment by RDS was feasible.

We used homophily scores to analyze the performance of RDS in recruiting diverse samples. Homophily, an index to evaluate whether the study sample from RDS could obtain a diverse sample [5], [8], demonstrated that RDS was an effective method to reach a diverse sample. The cross-recruitment and the related homophily implied that self-identified heterosexual MSM were not closely linked with the MSM networks of other orientations, possibly because most MSM who identified themselves as heterosexuals were money boys. The cross-recruitment among others was substantial, implying that recruitment chains did not become trapped within a single group, but instead, crossed group lines. This also meant that although the seeds were limited in some particular characteristics, the entire sample was not trapped within a particular group and we were successful in recruiting a diverse study population.

However, the large homophily (not close to zero) for venues for meeting sexual partners in our study also pointed out that our study still had the problem of oversampling of a certain group of hidden population, this was evidenced by the overestimate of the proportion of subjects who did usually meet their sexual partners through the internet (56.8%).

The distribution of the social network sizes of the participants changed significantly when we adjusted the crude distribution for the sizes of the social networks. Participants with strong network ties with other MSM were demonstrated by the large average social network sizes calculated according to sexual orientation. This might have indicated that social networks were important for distribution of the recruitment coupons and for continuation of recruitment. Therefore, social networks might be a very good medium for propagating HIV and STI prevention programs. The heterosexuals had the largest social network sizes, perhaps because most were money boys.

Our study was initially designed to have a sample size of 460 but only 430 were actually recruited. However, we found that the selected Deffs were nearly equal to or higher than 2, which demonstrated that the recruited sample size had met the needs.

Besides these, our study also found that a large proportion of our participants were engaged in unprotected anal intercourse (UAI), although majority of the participants had college level education. This result corroborated with our findings in a previous study [24]. Although these two observations seemed to be inconsistent with each other, this situation was common among MSM, and our previous study had already demonstrated that education and knowledge were poor predictors for UAI. This behavior was probably best predicted by intention and not associated with education or knowledge [25]. Other studies on sexually transmitted infections also reported similar findings [26].

RDS is usually feasible with the right choice of recruitment incentives, and the appropriate sampling size according to homophily and cross-states in the recruitment patterns. Only when the networks are independent, RDS is generally found to be unable to capture individuals from both populations. In our study, the situation seemed to be alike, which probably indicated that the choice of incentive worked nicely and the networks among our participants were not independent. The possibility of the speed of development of network in one group being faster than another existed among our study subjects as was evident from the difference in homophily across different groups. As the social network size was not extremely large, the estimates for the cross-groups probabilities were likely to be influenced. Our use of different seeds was somewhat likely to have taken care of this problem although the possibility of some residual effect was very much there.

We also recognized that our study data have many limitations. First, there was a possibility for information bias, especially recall bias. Secondly, although RDS was successful in studies of IDUs, FSWs and MSM in terms of recruitment efficiency in the past [10], [27]–[30], the data we collected were hard to analyze using conventional statistical software, particularly for univariate and bivariate analyses. We used RDSAT 5.6, which is a software designed specifically for analysis of data collected through RDS. An RDS sample without proper adjustment is nothing more than a very good snowball sample (not a representative sample). However, there might still be potential for bias due to over-sampling participants with large personal networks, which was avoided in our study by limiting numbers of distributed coupons during later waves. Also, our study was conducted in an STI clinic, which had the potential for introducing selection bias, and we think even RDS had partial capability for controlling this. To control and reduce the effects of some of those problems, we used the following methods. Four interviewers were trained for the interview to minimize interviewer bias while conducting the face-to-face interviews. We also appointed two persons to check for completion of questionnaires after each interview to minimize errors and inconsistencies. If any error was detected, it was corrected before the participant left the survey site. We believe that due to socio-behavioral issues for MSM, RDS was the optimal sampling method for our objectives. Further, we used the most advanced methods to adjust the collected data.

### Conclusion

This was the first time RDS was used for sampling MSM in Jiangsu Province. Overall, we found that use of RDS among MSM in Nanjing was feasible and the recruited sample was diverse. Thus, RDS was found to be an effective strategy to achieve a diverse sample of MSM. RDS may be applicable in other cities in Jiangsu Province and other areas of China to gather data from MSM, CSWs and IDUs for serologic and behavior surveillance in future.

## Supporting Information

Appendix S1 **The sample size and power estimation.**
(DOCX)

Appendix S2 **Relations between the sample size, Deff and standard error.**
(DOCX)
