# Supplementary material for: Feasibility of Recruiting a Diverse Sample of Men Who Have Sex with Men: Observation from Nanjing, China
Source: PLoS One. 2013 Nov 14;8(11):e77645. doi: 10.1371/journal.pone.0077645 (PMC3828322; doi:10.1371/journal.pone.0077645)
Supplement: Appendix S1 — The sample size and power estimation. (DOCX) [file pone.0077645.s001.docx]

**Appendix S1**

Appendix A summarizes the sample size and power estimation process in details. The estimation process was based on the following formula and assumptions:

In this formula:

Deff = design effect of 2.0 (recommendations vary from 1.25 to 2.0, therefore 2.0 was conservative);

$P_{A,1}$= the estimated proportion of sexual risk behavior at baseline (we used proportion of MSM having two or more partners as the proxy for sexual risk behavior and used 0.7 as the value for this according to recent surveys in China. This proportion had also been used for projecting the “at risk” sizes among MSM population of China);

$P_{A,2}$= the estimated proportion of sexual risk behavior at some different time after the baseline survey, so that (P2 - P1) was assumed as the magnitude of change we wanted to be able to detect (we assumed$P_{A,2}$=0.8 based on the rationale that we wished to be able to detect a 10% change in the proportion of MSM with having two or more partners);

$Z_{1-\frac{\alpha}{2}}$= the z-score corresponding to desired confidence level (we used the 95% confidence level and corresponding two-sided z-score);

$Z_{1-\beta}$= the z-score corresponding to the desired power (we used 80% power and the corresponding two-sided z-score)

The above parameters produced a required sample size of 460 participants per survey year, so, the estimated sample size for this study was 460.
