# Supplementary material for: Feasibility of Recruiting a Diverse Sample of Men Who Have Sex with Men: Observation from Nanjing, China
Source: PLoS One. 2013 Nov 14;8(11):e77645. doi: 10.1371/journal.pone.0077645 (PMC3828322; doi:10.1371/journal.pone.0077645)
Supplement: Appendix S2 — Relations between the sample size, Deff and standard error. (DOCX) [file pone.0077645.s002.docx]

***Appendix S2***

Appendix B summarizes the relations between the sample size, Deff, standard error, variance using RDS=$V(RDS,\mathop{P}\limits^{\wedge} _{A})$and variance while using simple random sampling=$V(SRS,\mathop{P}\limits^{\wedge} _{A})$, based on the calculation formula provided by Matthew J. Salganik and U.S. Department of Health and Human Service [25-27]:

.

Before we began this survey, the sample size estimation was based on equation 1, and the estimated sample size was 460, while the estimated design effect was 2. However, only 430 MSM were actually recruited, raising the concern whether the sample size met the requirements or not. To evaluate this, we used the following:

First, we designed $V(RDS,\mathop{P}\limits^{\wedge} _{A})$and $V(SRS,\mathop{P}\limits^{\wedge} _{A})$ to be determined using the formula below:

$$V(RDS,\mathop{P}\limits^{\wedge} _{A})=deff\cdot$$

$$V(SRS,\mathop{P}\limits^{\wedge} _{A})=$$

Next we calculated $V(SRS,\mathop{P}\limits^{\wedge} _{A})$for different variables and after analysis in RDSAT 5.6, we calculated the confidence intervals of the target variables, standards errors (se) and $V(RDS,\mathop{P}\limits^{\wedge} _{A})$using formula: and $V(RDS,\mathop{P}\limits^{\wedge} _{A})=deff\cdot$.

Finally, we used formula: and obtained the final Deff, and compared the estimated Deff and the observed Deff [25-27].

Using HIV positivity as an example,$V(SRS,\mathop{P}\limits^{\wedge} _{A})$, with the formulation of $V(SRS,\mathop{P}\limits^{\wedge} _{A})=\frac{0.066*(1-0.066)}{430}=0.00014$. Using RDSAT 5.6, we obtained the HIV positivity rate = 6.6%, its 95% confidence intervals being (2.9%, 10.3%), *se*=0.137, $V(RDS,\mathop{P}\limits^{\wedge} _{A})=$0.00036 and the final observed 2.48.
